# The Impact of Two Different Cold-Extruded Feeds and Feeding Regimens on Zebrafish Survival, Growth and Reproductive Performance

**DOI:** 10.3390/jdb6030015

**Published:** 2018-06-21

**Authors:** Joana F. Monteiro, Sandra Martins, Matheus Farias, Telma Costa, Ana Catarina Certal

**Affiliations:** Fish Platform, Champalimaud Center for the Unknown, 1400-038 Lisbon, Portugal; sandra.martins@neuro.fchampalimaud.org (S.M.); matheus.farias@neuro.fchampalimaud.org (M.F.); telma.costa@iol.pt (T.C.)

**Keywords:** zebrafish, dietary regimen, rotifers, feeds, survival, growth, reproductive performance

## Abstract

Zebrafish (*Danio rerio*) is one of the top model organisms used in biomedical research. Therefore, it is fundamental that zebrafish facilities continuously improve husbandry methods to provide fish with the best physiological and welfare conditions that suit each experimental purpose. Nutrition is a husbandry aspect that needs further optimization, as it greatly affects growth, reproduction, health and behaviour. Here, we have compared the impact of different feeding regimens on zebrafish survival, growth and reproductive performance. Mutant and wild-type zebrafish were raised using several combinations of two cold-extruded processed feeds—Skretting^®^GemmaMicro and Sparos^®^Zebrafeed—and one live feed (rotifers). Zebrafeed^®^ outperformed GemmaMicro^®^ in terms of survival rate, and embryo viability was also higher when the spawners were fed with Zebrafeed^®^ either from larval stage or upon sexual maturation. In contrast, GemmaMicro^®^ favoured growth, both in size and weight. The use of rotifers until 60 days post-fertilization improved survival of fish co-fed with GemmaMicro^®^, while delaying their growth. Zebrafeed^®^ performance was not affected by co-feeding rotifers. Overall, we showed that different nutritional formulas affect physiological parameters, allowing for the establishment of feeding protocols adapted to the objectives of each facility. At the same time, we validated Skretting^®^GemmaMicro and Sparos^®^Zebrafeed as two commercially available feeds that are well suited for zebrafish nutrition in a laboratory environment.

## 1. Introduction

Research in different models has historically promoted the importance of nutrition in outcomes related to development, health, disease and responses to toxic compounds in food and the environment [1,2,3]. Yet, there is a striking absence of literature showing the role of nutrition as an important regulator of health and disease in zebrafish. If nutrition is to be accepted as a critical part of health management, it is imperative to consider the typical end goal of fish production in a research setting: to provide fish that are clinically healthy and representative of animals exhibiting normal physiology [4]. Although the known nutritional requirements of other cyprinid fish can be an acceptable approach to zebrafish requirements [5,6], this does not take into account some particularities of the species, such as high growth rate, infinite growth, continuous egg production after maturation. The feeds and feeding regimens implemented by research laboratories for rearing zebrafish are varied [7]. In some cases, feeds and feeding regimens are implemented without a formal evaluation [8]. While the world aquaculture relies on fish meal (FM) as the main protein source in aquafeeds [9], research over the last decade has resulted in a significant decrease in the portion of FM in fish feeds, and there are species-specific limits in the FM replacement rate by, for example, soybean (SBM), above which irreversible damages occur affecting fish growth performance and welfare [10,11]. Therefore, meeting the specific nutritional requirements of zebrafish will potentiate growth and promote an optimal physiological status, minimizing undesirable effects and allowing for more consistent results [12]. The use of purified diets with defined ingredients facilitates the determination of the precise nutritional requirements, promoting optimal performance in fish stocks [13].

Zebrafish is an established model species and a powerful tool to study the modulation of reproductive processes through broodstock nutrition [14]. Proper management of broodstock nutrition encompasses providing the right amounts of the proper specialized nutrients, such as protein, essential fatty acids, vitamin E, vitamin C, carotenoids and phosphoglycerides during the different developmental stages, optimizing gonadal development and fecundity [15,16,17,18,19,20,21,22,23].

Having alternative suppliers of live and processed feeds, which results in fish with high, optimized and standardized growth, survival and breeding rates, is a management requisite for zebrafish facilities. Comparing different feeds tested in previous studies [14,24] that have demonstrated good impacts on growth and reproductive performance is the first step towards achieving this. This study aimed to understand which combination of commercial processed feeds, Skretting^®^GemmaMicro^®^ and/or Sparos^®^Zebrafeed^®^, complemented with Type “L” saltwater live rotifers *Brachionus plicatilis*, is more suitable for improved survival, growth and reproductive performance.

## 2. Materials and Methods

### 2.1. Zebrafish Strains and Embryo Generation

In this study, we used two strains of zebrafish that are widely used in biomedical research: wild-type AB and non-pigmented Nacre mutant (*mitfa* −/−). Embryos were obtained by overnight crossing 8–10 females with 4–6 males of each strain stock in 1.7 L sloping breeding tanks (Tecniplast^®^). The housing and husbandry of all animals were performed as published [25], and dietary adaptations were in accordance with the specifications of the present study (Table 1). Briefly, all embryos were collected, placed in 90 mm diameter petri dishes with standard embryo medium [26] and kept at 28 °C in an incubator with a photoperiod of 14 h:10 h/light:dark up to 5 days post-fertilization (dpf). Early 6 dpf larvae were transferred to 3.5 L tanks at a density of 35 fish/tank, each containing 400 mL of fish water and 100 mL of type “L” saltwater rotifers (*Brachionus plicatilis)* solution (0.5 mL of RG Complete™, ~800–900 rotifers/mL, conductivity ~3 ppt). At 8 dpf, water flow was started through the 3.5 L tanks, and larvae began to be fed according to their experimental group (Table 1).

### 2.2. Experimental Dietary Groups

Five experimental dietary groups were designed using different combinations of processed feeds (Skretting^®^GemmaMicro^®^; Sparos^®^Zebrafeed^®^) and a live feed (type “L” saltwater rotifers, *B. plicatilis*) (Table 1). The nutritional compositions of the processed feeds are described in Table 2. For each dietary group, there were 4 replicate tanks per strain. The size of the processed feed particles varied according to the fish developmental stage—using a seed dispenser, we provided Skretting^®^Gemma Micro^®^ 150, 300 or 500 or Sparos^®^Zebrafeed^®^ 100–200, 200–400 or 400–600 to animals at ages <30 dpf, 30–90 dpf and >90 dpf, respectively. Both dry feeds are easily distinguished by their colour—GemmaMicro^®^ grains are green, whereas Zebrafeed^®^ particles are orange red. Rotifer cultures were maintained as described [25], including feeding with refrigerated algae (RGcomplete^®^; Reed Mariculture). Regardless of the dietary regimen, all fish were fed 4×/day between 8 dpf and 60 dpf, 3×/day from 60 to 90 dpf and 2×/day from 90 dpf onwards. On weekends and holidays, animals were fed 1×/day with the processed feed of the corresponding experimental group. All tanks were given a similar volume of rotifer solution and a similar amount of processed feed, always in excess so that the feed quantity would not be a limiting factor. In order to avoid the impact of survival on growth, fish density/tank was readjusted at 30 dpf within each dietary group. To achieve this, fish from tanks with higher density (higher survival) were transferred to tanks with lower density (lower survival) resulting in a similar number of fish/tank.

### 2.3. Fish Survival Rate

The total number of fish in each tank was counted at the end of larval stage (30 dpf) and during adulthood (90 dpf), by manually transferring animals one by one to new tanks with a fishing net. The survival rates for each dietary treatment were calculated at 30 and 90 dpf as the total number of living fish in each dietary treatment per total number at the beginning of the experimental assay.

Fish welfare was checked twice/day by visual inspection for behavioural and/or physical abnormalities [27]. Carcasses were removed immediately upon detection and discarded as described [25].

### 2.4. Growth

Fish growth was examined using fork length (linear measurement) and dry weight (mass measurement). Both parameters were evaluated at 30 dpf and 90 dpf, using one tank with Nacre fish and another with AB fish for each dietary group. To determine the fork length, all fish were anesthetized with 0.16 g/L Tricaine (MS-222), photographed with a SONY^®^ SteadyShot (DSC-W510) and measured from snout to tail split using ImageJ software (v. 1.45s, Wayne Rasband, National Institutes of Health, Bethesda, MD, USA). To estimate the dry weight, fish were euthanized with 4 g/L Tricaine and individually stored in 1.5 mL microcentrifuge plastic tubes at −20 °C. They were then lyophilized for 48 h, and the dried residue was weighed on a precision scale.

### 2.5. Fish Sex Ratio

Fish were individually sexed at 90 dpf based on body shape and colour [28,29]. Briefly, males are slimmer and have pinky-blue streaks (in AB strain) or a yellowish colour (in Nacre strain). On the other hand, females have a protruding belly and silvery-blue streaks (in AB strain) or a pink colour (in Nacre strain). Only animals with clear sexual dimorphism were considered. The sex ratio for each dietary group was calculated as the percentage of males/females in each tank.

### 2.6. Reproductive Performance

To evaluate the impact of the five dietary regimens on embryo viability, all fish from each treatment were crossed at ≥90 dpf, in groups of 8–10 females and 4–6 males from each dietary treatment, and placed overnight in 1.7 L sloping breeding tanks (Tecniplast^®^). This procedure was repeated three times, on a weekly basis. Upon spawning, embryos from each cross were collected and distributed in 90 mm diameter Petri dishes at a density of ~50 embryos/plate. All unfertilized eggs were counted and removed, and plates were incubated at 28 °C. After 24 h, the numbers of live and non-viable embryos were counted. The embryo viability rate was calculated as the percentage of live embryos per total number of embryos.

Both Zebrafeed^®^ and GemmaMicro^®^ have been associated to good reproductive performances [14,30], but the two had never been compared. To assess if the use of Zebrafeed^®^ or GemmaMicro^®^ around the time of sexual maturation was enough to produce an effect on reproduction, we designed our experimental protocol to include two dietary groups where the only difference was the dry feed from 60 dpf onwards—both consumed GemmaMicro^®^ as the primary feed up to 60 dpf, but from then onwards group 2 was fed GemmaMicro^®^ and group 5 was fed Zebrafeed^®^. The fertilization rate and sperm viability were assessed for both groups. For the former, a new set of weekly incrosses was performed. Embryos and unfertilized eggs were counted at 7 hpf, and live embryos were gently transferred to a clean embryo medium-filled plate. The fertilization rate was calculated as the percentage of embryos per total number of eggs. At 24 hpf, live and non-viable embryos were counted and used to calculate embryo viability.

Sperm viability is often evaluated using several parameters, such as morphology, membrane integrity, ability to bind oocytes and motility, the latter being the most widely used assay. However, fertilization is considered to be the most informative parameter [31]. Therefore, sperm viability was assessed through the rate of in vitro fertilization using wild type oocytes and sperm from males of dietary groups 2 and 5. In particular, 8 rounds of in vitro fertilization (1 round every 2 weeks) were performed. Briefly, oocytes from 5–10 month old AB and Tübingen (TU) females under the normal feeding regimen [31] were collected by gentle squeezing. Each egg clutch was divided into two petri dishes and held in Aquaboost^®^ Ovacoat extender (Cryogenetics, USA). Sperm collected from males of dietary group 2 or 5 was added to each half of the egg clutch. After mixing, fish water was added to activate gametes for fertilization. At 24 hpf, the fertilization rate was assessed as described above. The survival and malformation rates (defined as larvae viability rate) were evaluated between 6 and 7 dpf.

### 2.7. Statistical Analysis

All data was analysed with the IBM SPSS Statistics software (v. 23, IBM Corp., Chicago, IL, USA), and all statistical analysis were based on widely established rules [24]. Specifically, after assumption verification (normality and homoscedasticity), Student *t*-tests and one-way ANOVA tests were used. When significant differences were observed, they were analysed with the Tukey’s HSD and DSM test in order to perceive which groups differed. For groups where assumptions were not verified, the Wilcoxon–Mann–Whitney and Kruskall–Wallis tests were used followed by pairwise comparison. Results were considered statistically significant for *p*-values < 0.05. Data sets where assumptions were verified were graphically presented in histograms with means and standard deviations. The remaining data sets were presented in boxplots with medians, interquartile ranges, maximums and minimums. Groups with statistically significant differences were graphically identified with a horizontal bar with small, perpendicular dashes on the top of each group, and the *p*-value level was represented on the top of the group that differs from the others, as follows: * *p* < 0.05, ** *p* < 0.01 and *** *p* < 0.0001.

## 3. Results

The initial comparisons between the AB and Nacre fish within each experimental treatment group returned no statistically significant differences between the two strains (*p*-value > 0.05, data not shown), suggesting that fish with different genetic backgrounds respond in the same way to each dietary regimen. Therefore, data from both strains were pooled and treated together to test for comparisons between the five dietary groups.

### 3.1. Survival Rate

**Figure 1:**
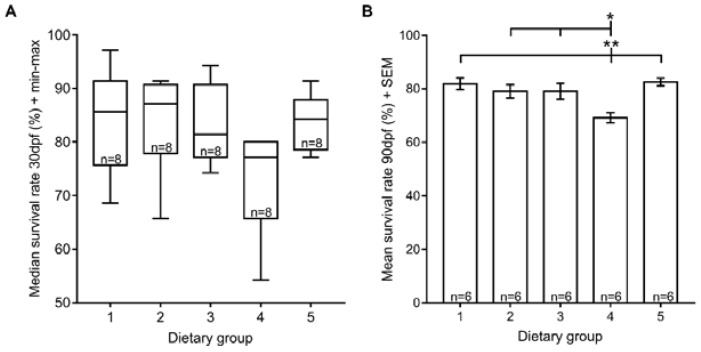
Zebrafish survival rates under the five different dietary regimens. (**A**) Median survival rate at 30 dpf; (**B**) mean survival rate at 90 dpf. Statistically significant differences in survival rates depicted: * *p* < 0.05, ** *p* < 0.01. Dietary Groups (Zeb = Zebrafeed^®^; Gem = GemmaMicro^®^; Rot = rotifers): group 1, Zeb. + Rot. <60 dpf, Zeb. >60 dpf; group 2, Gem. + Rot. <60 dpf, Gem. >60 dpf; group 3, Zeb. + Rot. <15 dpf, Zeb. >15 dpf; group 4, Gem. + Rot. <15 dpf, Gem. >15 dpf; group 5, Gem. + Rot. <60 dpf, Zeb. >60 dpf.

In this study, the larval median survival rate at 30 dpf ranged from 77.1% to 87.1%, and there were no significant differences among the dietary groups tested (Figure 1A). Nevertheless, at 90 dpf, fish fed exclusively with GemmaMicro^®^ from 15 dpf onwards (dietary group 4) had statistically significant lower survival rates than the remaining dietary groups (Figure 1B). On the other hand, fish that ate only Zebrafeed^®^ from 15 dpf (dietary group 3) had survival rates similar to dietary regimens that included rotifers up to later developmental stages (dietary groups 1, 2 and 5).

### 3.2. Growth

**Figure 2:**
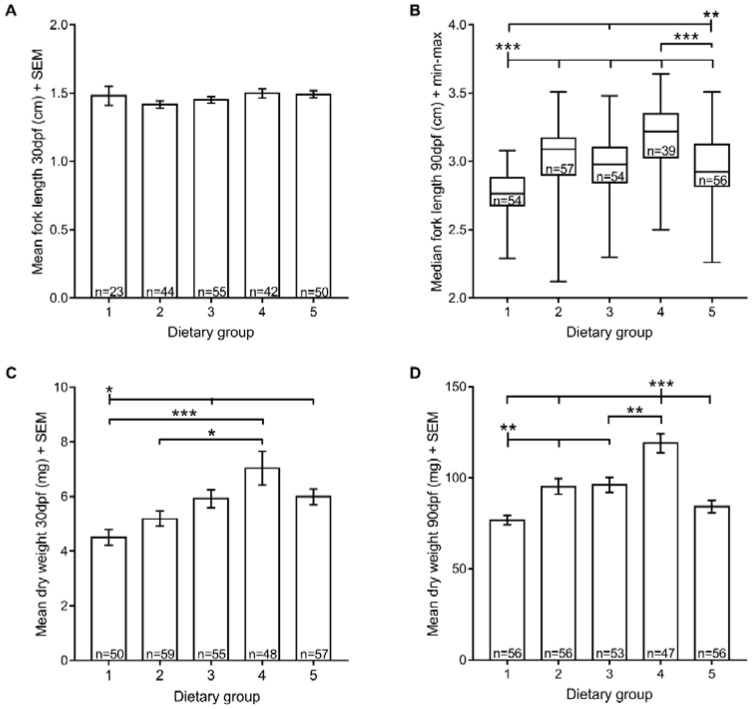
Zebrafish growth (size and weight) under five different feeding regimens. (**A**) Mean fork length at 30 dpf; (**B**) median fork length at 90 dpf; (**C**) mean dry weight at 30 dpf; (**D**) mean dry weight at 90 dpf. Statistically significant differences in size and weight are depicted: * *p* < 0.05, ** *p* < 0.01, *** *p* < 0.001. Group 1, Zeb. + Rot. <60 dpf, Zeb. >60 dpf; Group 2, Gem. + Rot. <60 dpf, Gem. >60 dpf; Group 3, Zeb. + Rot. <15 dpf, Zeb. >15 dpf; Group 4, Gem. + Rot. <15 dpf, Gem. >15 dpf; Group 5, Gem. + Rot. <60 dpf, Zeb. >60 dpf (Zeb = Zebrafeed^®^; Gem = GemmaMicro^®^; Rot = rotifers).

Organismal growth is the most commonly used metric to estimate nutrient requirements. Here, we analysed two growth measurements: fork length and dry weight. Regarding the fork length, all fish exhibited similar sizes at 30 dpf (Figure 2A). However, at 90 dpf, fish fed with both Zebrafeed^®^ and rotifers until 60 dpf (dietary group 1) were smaller than all others (fork length: 2.77 ± 0.15 cm). On the contrary, a diet composed exclusively of GemmaMicro^®^ from 15 dpf (dietary group 4) was associated with fish with higher fork length than all diets that included Zebrafeed^®^ (dietary groups 1, 3 and 5) (Figure 2B).

Regarding the dry weight, we found a similar pattern. Fish raised on Zebrafeed^®^ and rotifers until 60 dpf (dietary group 1) weighed less than the remaining experimental groups, both at 30 and 90 dpf, with a few exceptions (dietary group 2 at 30 dpf; dietary group 5 at 90 dpf) (Figure 2C,D). Conversely, at 30 dpf, animals raised exclusively using GemmaMicro^®^ from 15 dpf onwards (dietary group 4) weighed more than when rotifers were still provided (dietary groups 1 and 2), and at 90 dpf, the same group had heavier fish than all others (Figure 2C,D). In fact, dietary group 1 weighed significantly less than dietary group 5 at 30 dpf when the two were provided with different processed feeds (Figure 2C), but they were not significantly different at 90 dpf, a timepoint at which both groups had been fed with the same dry feed (Zebrafeed^®^) for at least one month (Figure 2C). This not only agrees with GemmaMicro^®^ yielding higher growth rates than Zebrafeed^®^, but it also suggests that changing processed feds at late developmental stages produces significant effects in a time period as short as one month.

### 3.3. Sex Ratio

**Figure 3:**
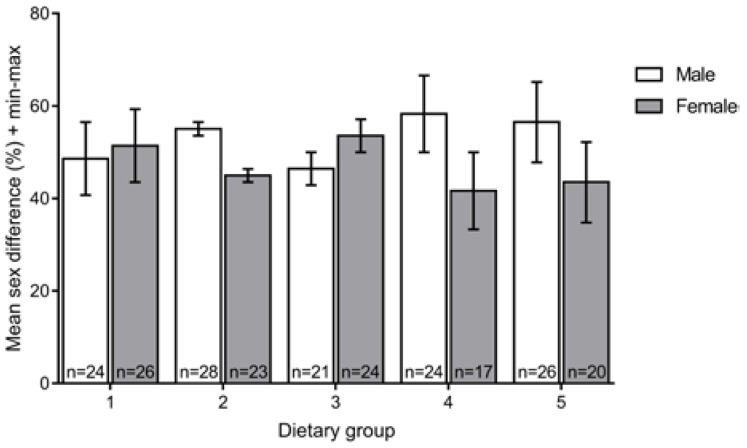
Mean sex ratio for each dietary group at 90 dpf. No statistically significant differences were found among the five dietary regimens. Group 1, Zeb. + Rot. <60 dpf, Zeb. >60 dpf; Group 2: Gem. + Rot. <60 dpf, Gem. >60 dpf; Group 3, Zeb. + Rot. <15 dpf, Zeb. >15 dpf; Group 4, Gem. + Rot. <15 dpf, Gem. >15 dpf; Group 5, Gem. + Rot. <60 dpf, Zeb. >60 dpf (Zeb = Zebrafeed^®^; Gem = GemmaMicro^®^; Rot = rotifers).

To determine if the different feeds affected sex differentiation, we compared the numbers of males and females in the different experimental groups at 90 dpf and did not observe significant differences (U = 357.2; *p* = 0.328) (Figure 3).

### 3.4. Reproductive Performance

**Figure 4:**
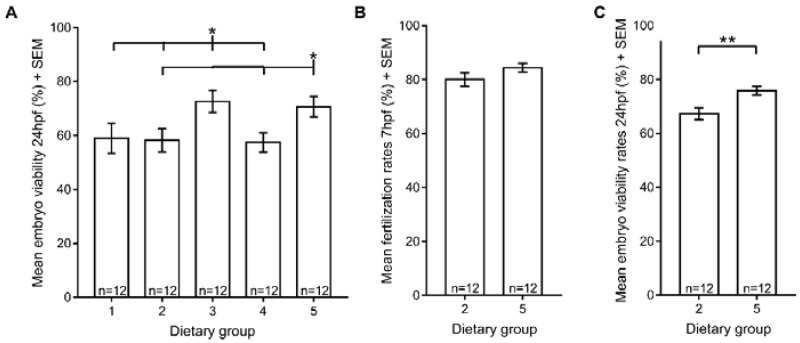
Reproductive performance is affected by processed feeds but not by rotifers. (**A**) Mean embryo viability at 24 hpf; (**B**) mean fertilization at 7 hpf for dietary groups 2 and 5; (**C**) mean embryo viability at 24 hpf for dietary groups 2 and 5. Statistically significant differences in reproductive parameters are depicted: *p < 0.05, ** *p* < 0.01, *** *p* < 0.001. Group 1, Zeb. + Rot. <60 dpf, Zeb. >60 dpf; Group 2, Gem. + Rot. <60 dpf, Gem. >60 dpf; Group 3, Zeb. + Rot. <15 dpf, Zeb. >15 dpf; Group 4, Gem. + Rot. <15 dpf, Gem. >15 dpf; Group 5, Gem. + Rot. <60 dpf, Zeb. >60 dpf (Zeb = Zebrafeed^®^; Gem = GemmaMicro^®^; Rot = rotifers).

A positive correlation between Zebrafeed^®^ and reproductive performance has been previously reported [14]. Therefore, we tested several reproductive parameters, starting with the viability of embryos from all five experimental dietary regimens. Our results showed that extending the period of rotifers co-feeding from 15 dpf to 60 dpf did not improve embryo viability (Figure 4A), yet processed feeds affected zebrafish reproductive function. Specifically, embryo viability was higher when spawners were fed exclusively with Zebrafeed^®^ from the larval stage (15 dpf) or when Zebrafeed^®^ replaced GemmaMicro^®^ at 60 dpf, which is around the time that fish reach sexual maturation. Moreover, in dietary groups fed with GemmaMicro^®^ and/or with more rotifer meals than processed meals (dietary groups 1, 2 and 4), embryo viability was lower. All these results suggest that Zebrafeed^®^ outperforms GemmaMicro^®^ in terms of embryo viability, as long as it is provided to the fish in sufficient amounts.

To further confirm the positive effect of Zebrafeed^®^ on zebrafish reproduction, we focused on dietary groups 2 and 5, because both groups were first raised on GemmaMicro^®^ from the larval stage, but upon sexual maturation (60 dpf), dietary group 2 was kept under GemmaMicro^®^, whereas group 5 was switched to Zebrafeed^®^. After a new round of crosses using these fish, the fertilization and embryo viability rates were analysed. Our results showed no differences between fertilization rates of the two groups (Figure 4C). In contrast, embryo viability was higher in dietary group 5, where Zebrafeed^®^ replaced GemmaMicro^®^ at 60 dpf (Figure 4B), reinforcing that Zebrafeed^®^ outperforms GemmaMicro^®^ in terms of reproductive performance.

**Figure 5:**
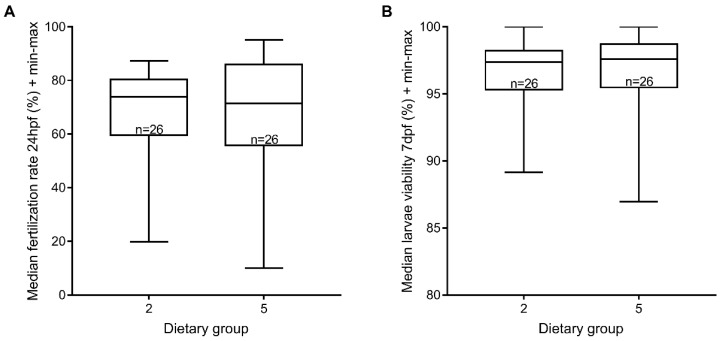
Sperm viability was not affected by processed feeds. (**A**) Median in vitro fertilization rate using sperm from males under GemmaMicro^®^ (dietary group 2) or Zebrafeed^®^ (dietary group 5) at 24 hpf; (**B**) corresponding larvae viability rate (6–7 dpf). There were no statistically significant differences, for either parameters. Group 1, Gem. + Rot. <60 dpf, Gem. >60 dpf; Group 5, Gem. + Rot. <60 dpf, Zeb. >60 dpf (Zeb = Zebrafeed^®^; Gem = GemmaMicro^®^; Rot = rotifers).

It has been reported that Zebrafeed^®^ increments sperm motility [14]. Taking that into account, we evaluated the sperm quality using sperm from males from dietary groups 2 and 5 in in vitro fertilization assays, and using the fertilization rate at 24 hpf and larvae viability rate at 6–7 dpf as proxies for sperm viability. We did not find any significant differences between the two processed feeds tested (Figure 5). Taken together, our results could not confirm that Zebrafeed^®^ improves sperm quality.

## 4. Discussion

In the Champalimaud Fish Platform, a feeding program comprising rotifers, *Artemia* and GemmaMicro^®^ (Skretting^®^) has been optimized and implemented [25] based on studies that suggested this is the best diet in terms of both larval rearing and breeding performance [30,32,33]. Importantly, the success of GemmaMicro^®^ compared to other dry feeds seems to be more associated with its production method [25], named “cold extrusion spheronizer agglomeration”, which better preserves nutrients, rendering them more digestible and stable in water [34,35,36]. Recently, a new processed feed using the same production method has become commercially available: Zebrafeed^®^ (Sparos^®^). As facilities need to constantly seek for new and improved solutions to maintain and breed fish, the present study was devised to compare the survival, growth and reproductive performance of zebrafish raised on either GemmaMicro^®^ and/or Zebrafeed^®^. In addition, we wanted to understand to which extent fish benefit from the use of rotifers as a co-feed to these processed feeds beyond the early larval stage (>15 dpf). Our results validate both GemmaMicro^®^ and Zebrafeed^®^ as two commercially available feeds that are well suited for laboratory zebrafish and show that the benefits of co-feeding rotifers after early larval stage vary depending on the processed diet used and considering the main research interests of each facility.

The first parameter assessed was survival rate, because it generally reflects a response to an extreme limitation or toxicity of a specific nutrient [37]. At 30 dpf, we obtained similar survival rates in all dietary groups, ranging from 77.1% to 87.1%. Even though this indicates that there is room for further nutritional improvements to meet zebrafish needs, one should keep in mind that some mortality can be associated with genetic factors inherent to the strain [38]. Thus, our data suggest that all of the dietary regimens tested seem to respect the nutritional requirements of zebrafish during early development to a similar and satisfactory extent.

However, as fish develop, their nutritional requirements change, and by 90 dpf, some of the dietary regimens had different efficiencies in terms of survival. In a previous study using GemmaMicro^®^ [30], survival rates under similar feeding conditions in our facility did not go beyond 41% by 90 dpf. In this study, by adding rotifers as a co-feed until 15 dpf before switching to a GemmaMicro^®^-only diet, we were able to increase survival rates from 41% to more than 65% at 90 dpf, demonstrating that the use of live feeds, in particular rotifers, in combination with this dry feed during early larval development greatly improves survival rate, even in later developmental stages, as previously suggested [30,32,39,40]. Moreover, extending the period of GemmaMicro^®^ and rotifer co-feeding to 60 dpf increased the survival rate even further which could be related to the fact that larvae and juveniles feed most efficiently when they are given the opportunity to capture small, slow moving prey that move within their field of vision [33]. On the other hand, Zebrafeed^®^ alone accounted for higher survival than GemmaMicro^®^ and was able to replace the use of rotifers as a co-feed from as early as 15 dpf, revealing that Zebrafeed^®^ could be a better choice than GemmaMicro^®^ for facilities that need to rely only on processed feeds [32,39].

When it comes to growth performance, comparisons with previous studies are not easy due to the lack of uniformity in rearing conditions and evaluated parameters, such as growth measurements and developmental time points analysed. Even so, many studies have suggested that a mixed diet composed of live and processed feeds or live feeds alone accounts for higher growth [40,41,42,43]. Accordingly, at 90 dpf, we obtained higher fork lengths for fish raised on GemmaMicro^®^ and also rotifers up to 15 dpf (dietary group 4) than what has been described for fish reared on GemmaMicro^®^ alone [30]. Nevertheless, increasing the period of rotifer feeding from 15 to 60 dpf did not increase growth. This could have been caused by ingestion of non-equivalent amounts of rotifers and dry feed and consequently, different nutritional intakes [44], considering that we provided live and dry feeds until apparent satisfaction. In the future, it would be interesting to measure the biomass of the live and dry feeds and provide equivalent quantities. Another explanation for our results is that the first feeding stage (~8–15 dpf) could be a crucial time window for rotifer feeding, during which, nutrition may condition behaviour and physiological processes to such an extent that could affect growth in later developmental stages [32]. After that initial stage, a diet composed exclusively of processed feeds accounts for the increased growth, likely because it allows for greater control in terms of quantity and nutritional quality [45].

Comparing the growth performances of the dry feeds, GemmaMicro^®^ outperformed Zebrafeed^®^ both in length and weight. In addition, we detected a decrease in growth one month after switching feeds from GemmaMicro^®^ to Zebrafeed^®^, further supporting the proposal that GemmaMicro^®^ promotes higher growth than Zebrafeed^®^. Accordingly, previous studies comparing the growth performances of different dry feeds all agreed that GemmaMicro^®^ sustains faster growth rates [14,30,45]. However, one should keep in mind that in our study, the faster growth rates of fish fed only with GemmaMicro^®^ from 15 dpf (dietary group 4) may have been influenced by their lower survival rate and consequently, lower fish density, as the latter is a factor that is known to increase growth [30,37]. Furthermore, we noticed that, although not statistically significant, the dietary groups that included GemmaMicro^®^ were slightly male-biased, whereas those that ate Zebrafeed^®^ appeared to be more female-biased, which is an apparent benefit for facility management purposes where a higher female/male ratio is usually required. What determines the sexual ratio is still under debate, but it has been related to different fish stocking densities [38]. Our sex ratio results are in contrast with the larger sizes and weights obtained with GemmaMicro^®^ which would be more likely to be related with more females, so it would be interesting to perform a relative growth analysis for fish raised with each feed as it could be the case that Zebrafeed^®^ has a slower growth curve but would match the size of GemmaMicro^®^-fed fish within a few days [46,47]. In fact, size is usually more associated with sexual maturity than with age [48], and no delay in sexual maturation in fish raised with Zebrafeed^®^ as compared to GemmaMicro^®^ was observed.

Regarding reproductive performance, both sperm viability and fecundity were similar in all experimental dietary groups. Even though we could not confirm a direct benefit of Zebrafeed^®^ on sperm quality, as previously described [14], it should be taken into account that the parameters used to analyse sperm quality on each study were not the same. In addition, we found that fish fed with Zebrafeed^®^ more than once a day produced embryos with higher viability, which could be a long-term effect of diet-related sperm quality [49]. This higher embryo viability was observed not only for spawners that ate exclusively Zebrafeed^®^ since larval stages (15 dpf) but also for fish fed with Zebrafeed^®^ only from 60 dpf onwards, i.e., around the time they became sexually mature. This suggests that Zebrafeed^®^ is capable of improving reproductive performance in a short time frame after changing feeds which we propose could be related to the specific lipid composition of Zebrafeed^®^ [14]. Even though the crude lipid percentages of GemmaMicro^®^ and Zebrafeed^®^ are similar (Table 2) and a detailed lipid composition is not available for the two feeds, it has been shown that feeding zebrafish spawners with a diet enriched in essential polyunsaturated fatty acids (PUFA) is enough to increase the quality of gametes and larvae in as little as 14 days of change in feeding [50].

Several studies have made it clear that the presence of essential fatty acids (especially PUFAs) as well as several vitamins (vitamin A, C, E among others) in adequate proportions is required to ensure normal egg and embryo viability and normal larval development [15,19,42,49,50,51], even though the exact values for zebrafish are yet to be determined. In fact, adequate nutrition is fundamental for most physiological functions, not only affecting directly survival, growth and reproduction, but also influencing several other processes, including endocrine, neurological and immunological functions [39]. The two processed feeds compared here were manufactured through the same production method and have similar gross nutritional compositions (Table 2). Therefore, the dichotomy obtained here—higher growth for fish raised with GemmaMicro^®^ and better survival and embryo viability for fish fed with Zebrafeed^®^—should be related to different micronutrient proportions or to the way they become available to cells. The answer may lie in the ingredients used in each feed: GemmaMicro^®^ is based on fish meal and fish oil, whereas Zebrafeed^®^ is based on fish, squid and krill meal and on fish and krill oil. It would be interesting to investigate the exact effects of krill meal and oil on zebrafish rearing, as these small crustaceans have a very high nutritional level and represent the primary feed for many fish and marine mammals in the wild [44]. Another possible factor worth assessing is the role of visual attractiveness of these processed feeds related to their colour (Zebrafeed^®^ is orange red, GemmaMicro^®^ is green), as visual appeal is known to affect prey-capture behaviour [52].

In conclusion, we have validated different dietary regimens that can be successfully implemented in a research zebrafish facility setting, adapted to the research objectives. For instance, Zebrafeed^®^ seems to meet zebrafish demands more closely than GemmaMicro^®^ in terms of survival and reproductive performance which could benefit facilities that prioritize the capacity of maximizing the number of animals and embryo production (e.g., for drug or mutagenesis screens). On the other hand, GemmaMicro^®^ promotes higher growth rates, which could be useful when the biggest concern is to minimize the time animals take to reach a certain size of interest or to reduce the life cycle (e.g., for quick high-throughput transgenic screens or juvenile studies). Interestingly, other dietary regimens tested in previous studies have also concluded that the feeding regimes that are most conductive to growth do not necessarily maximize reproductive success in this species [45]. Regarding the use of rotifers, we confirmed that their use during the first stages of exogenous feeding improves larval rearing. However, the benefits of extending the use of rotifers until later developmental stages are not so straightforward, and their use should be considered in light of other factors, including the processed feeds used and the scientific goals of the fish. For instance, rotifers can be an important co-feed in facilities that serve research communities focused on behavioural studies, because live feeds ensure the maintenance of zebrafish natural prey-capture behaviour.

We believe this study brings new data and insights that will be useful for designing and implementing feeding programs in zebrafish facilities in accordance with their research programs.

## Figures and Tables

**Table 1:** Experimental dietary groups, starting at 8 days post-fertilization (dpf). The number of daily meals of each feed is listed as well as the size of the grains of processed feeds.

	Age (dpf)	Skretting^®^GemmaMicro	Sparos^®^Zebrafeed	Live Feed
**Experimental Dietary Group 1**	8–15	-	1× 100–200	3× Rotifers
	15–30	-	1× 100–200	3× Rotifers
	30–60	-	1× 200–400	3× Rotifers
	60–90	-	3× 200–400	-
	>90	-	2× 400–600	-
**Experimental Dietary Group 2**	8–15	1× 150	-	3× Rotifers
	15–30	1× 150	-	3× Rotifers
	30–60	1× 300	-	3× Rotifers
	60–90	3× 300	-	-
	>90	2× 500	-	-
**Experimental Dietary Group 3**	8–15	-	1× 100–200	3× Rotifers
	15–30	-	4× 100–200	-
	30–60	-	4× 200–400	-
	60–90	-	3× 200–400	-
	>90	-	2× 400–600	-
**Experimental Dietary Group 4**	8–15	1× 150		3× Rotifers
	15–30	4× 150		-
	30–60	4× 300		-
	60–90	3× 300		-
	>90	2× 500		-
**Experimental Dietary Group 5**	8–15	1× 150	-	3× Rotifers
	15–30	1× 150	-	3× Rotifers
	30–60	1× 300	-	3× Rotifers
	60–90	-	3× 200–400	-
	>90	-	2× 400–600	-

**Table 2:** Nutritional composition of the processed feeds tested. The nutritional content was the same for all grain sizes. Information was gathered from the products’ labels.

	Skretting^®^ Gemma Micro (%)	Sparos^®^ Zebrafeed (%)
**Protein**	59	63
**Fat and Oils**	14	14
**Fiber**	0.2	1.8
**Ash**	14	12
**Phosphorus**	1.3	2.3
**Main ingredients**	Fish meal, lecithin, wheat gluten, dried seaweed, fish oil, maize starch, vitamins and minerals	Squid meal, wheat gluten, fish meal, fish protein concentrate, krill meal, pea protein concentrate, starch, lecithin, krill oil, fish oil.

## References

[1] Barnard D.E., Lewis S.M., Teter B.B., Thigpen J.E. (2009). Open- and closed-formula laboratory animal diets and their importance to research. J. Am. Assoc. Lab. Anim. Sci..

[2] Harding J.D., Van Hoosier J.L., Grieder F.B., Hau J., Schapiro S.J. (2011). The contribution of laboratory animals to medical progress—past, present and future. Handbook of Laboratory Animal Science—Essential Principles and Practices.

[3] Hau J., Hau J., Gerald L. (2005). Animal models. Handbook of Laboratory Animal Science—Animal Models.

[4] Watts S.A., Lawrence C., Powell M., D’Abramo R.L. (2016). The Vital Relationship between Nutrition and Health in Zebrafish. Zebrafish.

[5] Lawrence C. (2007). The husbandry of zebrafish (*Danio rerio*): A review. Aquaculture.

[6] Kaushik S., Georgia I., Koumoundouros G. (2011). Growth and body composition of zebrafish (*Danio rerio*) larvae fed a compound feed from first feeding onward: toward implications on nutrient requirements. Zebrafish.

[7] Castranova D., Lawton A., Lawrence C., Baumann D., Best J., Coscolla J., Doherty A., Ramos J., Hakkesteeg J., Wang C. (2011). The effect of stocking densities on reproductive performance in laboratory zebrafish (*Danio rerio*). Zebrafish.

[8] Gonzales J. (2012). Preliminary evaluations on the growth and early reproductive performance of zebrafish (*Danio rerio*). J. Am. Assoc. Lab. Anim. Sci..

[9] (2010). SOFIA: The State of World Fisheries and Aquaculture.

[10] Geay F., Ferraresso S., Zambonino-Infante J.L., Bargelloni L., Quentel C., Vandeputte M., Kaushik S., Cahu C.L., Mazurais D. (2011). Effects of the total replacement of fish based diet with plant-based diet on the hepatic transcriptome of two European sea bass (Dicentrarchus labrax) half-sibfamilies showing different growth rates with the plant-based diet. BMC Genom..

[11] Sahlmann C., Sutherland B.J.G., Kortner T.M., Koop B.F., Krogdahl A., Bakke A.M. (2013). Early response of gene expression in the distal intestine of Atlantic salmon (*Salmo salar* L.) during the development of soybean meal induced enteritis. Fish Shellfish Immunol..

[12] Fernandes H., Peres H., Carvalho A.P. (2016). Dietary Protein Requirement during Juvenile Growth of Zebrafish (*Danio rerio*). Zebrafish.

[13] Lawrence C., William D.H. (2011). The Zebrafish: Genetics, Genomics and Informatics. Methods in Cell Biology.

[14] Diogo P., Martins G., Gavaia P., Pinto W., Dias J., Cancela L., Martınez-Páramo S. (2015). Assessment of nutritional supplementation in phospholipids on the reproductive performance of zebrafish, *Danio rerio* (Hamilton, 1822). Appl. Ichthyol..

[15] Rainuzzo J.R., Reitan K.I., Olsen Y. (1997). The significance of lipids at early stages of marine fish: A review. Aquaculture.

[16] Watanabe T., Cowley C.B., Mackie A.M., Bell J.K. (1985). Importance of the study of broodstock nutrition for further development of aquaculture. Nutrition and Feeding in Fish.

[17] Fernandez-Palacios H., Izquierdo M., Robaina L., Valencia A., Salhi M., Montero D. (1997). The effect of dietary protein and lipid from squid and fish meals on egg quality of broodstock for gilthead seabream (*Sparus aurata*). Aquaculture.

[18] Izquierdo M.S., Fernandez-Palacios H., Tacon A.G.J. (2001). Effect of broodstock nutrition on reproductive performance of fish. Aquaculture.

[19] Alsop D., Matsumoto J., Brown S., Van Der Kraak G. (2008). Retinoid requirements in the reproduction of zebrafish. Gen. Comp. Endocrinol..

[20] Jaya-Ram A., Kuah M.K., Lim P.S., Kolkovski S., Shu-Chien A.C. (2008). Influence of dietary HUFA levels on reproductive performance, tissue fatty acid profile and desaturase and elongase mRNAs expression in female zebrafish *Danio rerio*. Aquaculture.

[21] Miller G.W., Labut E.M., Lebold K.M., Floeter A., Tanguay R.L., Traber M.G. (2012). Zebrafish (*Danio rerio*) fed vitamin E-deficient diets produce embryos with increased morphologic abnormalities and mortality. J. Nutr. Biochem..

[22] Robinson B.D., Drew R.E., Murdoch G.K., Powell M., Rodnick K.J., Settles M., Stone D., Churchill E., Hill R.A., Papasani R.A. (2008). Sexual dimorphism in hepatic gene expression and the response to dietary carbohydrate manipulation in the zebrafish (*Danio rerio*). Comp. Biochem. Physiol. D-Genom. Proteom..

[23] Cabrita E., Robles V., Sarasquete C., Herráez M.P., Tiersch T., Mazik P.M. (2011). New insights on sperm quality analysis for the improvement of broodstock. Cryopreservation of Aquatic Species.

[24] James G., Witten D., Hastie T., Tibshirani R. (2013). Statistical learning. An Introduction to Statistical Learning: with Applications in R.

[25] Martins S., Monteiro J.F., Vito M., Weintraub D., Almeida J., Certal A.C. (2016). Toward an Integrated Zebrafish Health Management Program Supporting Cancer and Neuroscience Research. Zebrafish.

[26] Westerfield M. (2000). General Methods for zebrafish care. The Zebrafish Book. A Guide for the Laboratory Use of Zebrafish (Danio rerio).

[27] Zebrafish International Resource Center—Monitoring of Fish Morbidity and Mortality. http://zebrafish.org/documents/protocols/pdf/health_monitoring/daily_monitoring_fish_morbidity_07_2015.pdf.

[28] Darrow K.O., Harris W. (2004). Characterization and development of courtship in Zebrafish, *Danio rerio*. Zebrafish.

[29] Zebrafish in the Classroom. http://www.zfic.org/common%20techniques/Gender%20identification%20guide.pdf.

[30] Farias M., Certal A.C. (2016). Different Feeds and Feeding Regimens have an Impact on Zebrafish Larval Rearing and Breeding Performance. SOJ Aquat. Res..

[31] Yang H., Tiersch T.R. (2009). Current status of sperm cryopreservation in biomedical research fish models: Zebrafish, Medaka, and Xiphophorus. Comp. Biochem. Physiol. C Toxicol. Pharmacol..

[32] Best J., Adatto I., Cockington J., James A., Lawrence C. (2010). A novel method for rearing first-feeding larval zebrafish: polyculture with Type L saltwater rotifers (*Brachionus plicatilis*). Zebrafish.

[33] Lawrence C., James A., Mobley S. (2015). Successful Replacement of *Artemia salina nauplii* with Marine Rotifers (*Brachionus plicatilis*) in the Diet of Preadult Zebrafish (*Danio rerio*). Zebrafish.

[34] Boyaci B.B., Han J., Masatcioglu M.T., Yalcin E., Celik S., Ryu G., Koksel H. (2012). Analytical Methods—Effects of cold extrusion process on thiamine and riboflavin contents of fortified corn extrudates. Food Chem..

[35] Steel C.J., Leoro M.G.V., Schmiele M., Ferreira R.E., Chang Y.K., El-Sonbati A. (2012). Thermoplastic Extrusion in Food Processing. Thermoplastic Elastomers.

[36] New! TECH Feature: Cold Extrusion—Headline News. http://www.aquafeed.com/news/headline-news-article/601/NEW-TECH-FEATURE-COLD-EXTRUSION/.

[37] Watts S.A., Powell M., D’Abramo L.R. (2012). Fundamental approaches to the study of zebrafish nutrition. ILAR J..

[38] Wilson C. (2012). Aspects of larval rearing. ILAR J..

[39] Siccardi A.J., Garris H.W., Jones W.T., Moseley D.B., D’Abramo L.R., Watts SA. (2009). Growth and survival of zebrafish (*Danio rerio*) fed different commercial and laboratory diets. Zebrafish.

[40] Carvalho A.P., Araújo L., Santos M.M. (2006). Rearing zebrafish (*Danio rerio*) larvae without live food: Evaluation of a commercial, a practical and a purified starter diet on larval performance. Aquac. Res..

[41] Goolish E.M., Okutake K., Lesure S. (1999). Growth and Survivorship of Larval Zebrafish *Danio rerio* on Processed Diets. N. Am. J. Aquac..

[42] Gonzales J.M., Law S.H. (2013). Feed and feeding regime affect growth rate and gonadosomatic index of adult zebrafish (*Danio rerio*). Zebrafish.

[43] Singh S.K., Mishra U., Roy S.D., Chadha N.K., Venkateshwarlu G. (2012). Effect of feeding enriched formulated diet and live feed on growth, survival and fatty acid profile of Deccan Mahseer, Tor khudree (Sykes) first feeding fry. J. Aquac. Res. Dev..

[44] Schramm M.J. “Tiny Krill: Giants in Marine Food Chain”. NOAA National Marine Sanctuary Program. https://sanctuaries.noaa.gov/news/features/1007_krill.html.

[45] Lawrence C., Best J., James A., Maloney K. (2012). The effects of feeding frequency on growth and reproduction in zebrafish (*Danio rerio*). Aquaculture.

[46] Hopkins K.D. (1992). Reporting Fish Growth: A Review of the Basics. J. World Aquac. Soc..

[47] Gómez-Requeni P., Conceição L.E., Jordal A.E.O., Rønnestad I. (2010). A reference growth curve for nutritional experiments in zebrafish (*Danio rerio*) and changes in whole body proteome during development. Fish Physiol. Biochem..

[48] Harper C., Lawrence C., Suckow M.A. (2011). Husbandry. The Laboratory Zebrafish.

[49] Bobe J., Labbé C. (2010). Egg and sperm quality in fish. Gen. Comp. Endocrinol..

[50] Nowosad J., Kucharczyk D., Targońska K. (2017). Enrichment of Zebrafish *Danio rerio* (Hamilton, 1822) Diet with Polyunsaturated Fatty Acids Improves Fecundity and Larvae Quality. Zebrafish.

[51] Traber M.G., Atkinson J. (2007). Vitamin E antioxidant and nothing more. Free Radic. Biol. Med..

[52] Lawrence C., Sanders E., Henry E. (2012). Methods for culturing saltwater rotifers (*Brachionus licatilis*) for rearing larval zebrafish. Zebrafish.

